# Transcranial ultrasound stimulation of the human motor cortex

**DOI:** 10.1016/j.isci.2021.103429

**Published:** 2021-11-13

**Authors:** Yi Zhang, Liyuan Ren, Kai Liu, Shanbao Tong, Ti-Fei Yuan, Junfeng Sun

**Affiliations:** 1 Shanghai Key Laboratory of Psychotic Disorders, Shanghai Mental Health Center, Shanghai Jiao Tong University School of Medicine, Shanghai 200030, China; 2 School of Biomedical Engineering, Shanghai Jiao Tong University, Shanghai 200230, China; 3 Co-innovation Center of Neuroregeneration, Nantong University, Nantong, Jiangsu 226019, China; 4 Brain Science and Technology Research Center, Shanghai Jiao Tong University, Shanghai 200230, China; 5 Translational Research Institute of Brain and Brain-Like Intelligence, Shanghai Fourth People’s Hospital Affiliated to Tongji University School of Medicine, Shanghai, China

**Keywords:** Biological sciences, Neuroscience, Techniques in neuroscience

## Abstract

It has been 40 years since the report of long-term synaptic plasticity on the rodent brain. Transcranial ultrasound stimulation (TUS) shows advantages in spatial resolution and penetration depth when compared with electrical or magnetic stimulation. The repetitive TUS (rTUS) can induce cortical excitability alteration on animals, and persistent aftereffects were observed. However, the effects of rTUS on synaptic plasticity in humans remain unelucidated. In the current study, we applied a 15-min rTUS protocol to stimulate left primary motor cortex (l-M1) in 24 male healthy participants. The single-pulsed transcranial magnetic stimulation-evoked motor evoked potential and Stop-signal task was applied to measure the rTUS aftereffects. Here, we report that conditioning the human motor cortex using rTUS may produce long-lasting and statistically significant effects on motor cortex excitability as well as motor behavior, without harmful side effects observed. These findings suggest a considerable potential of rTUS in cortical plasticity modulation and clinical intervention for impulsivity-related disorders.

## Highlights

• rTUS induces motor cortex excitability alteration lasting for 30 min
• A 15-min rTUS over left M1 improves inhibitory control function
• rTUS has considerable potential in cortical plasticity modulation

## Graphical abstract

**Figure:**
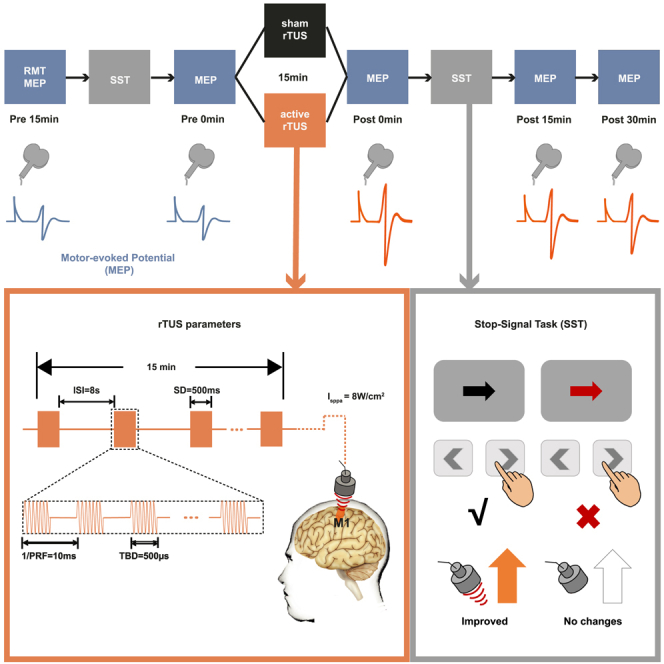

## Introduction

Synaptic plasticity represents one important cellular mechanism underlying learning and memory (Martin et al., 2000). Despite numerous studies probing and manipulating synaptic plasticity in live animals, investigation on human subjects remains relatively limited. Transcranial magnetic stimulation (TMS) and transcranial direct current stimulation provide noninvasive strategies to modulate cortical plasticity on the human brain, yet these approaches suffer from limitations in either spatial resolution or depth of stimulation (Krishna et al., 2018).

Transcranial ultrasound stimulation (TUS) has been an emerging approach to generate relatively focal activation in different brain regions (including deep regions), such as the frontal cortex (Fine et al., 2019, 2020; Verhagen et al., 2019), the primary visual cortex (V1) (Lee et al., 2016b), the temporal cortex (Hameroff et al., 2013), the somatosensory cortex (Lee et al., 2015; Legon et al., 2014; Mueller et al., 2014), caudate (Ai et al., 2016), hippocampus (Nicodemus et al., 2019), and thalamus (Legon et al., 2018a; Monti et al., 2016). Single-element TUS pulses could effectively activate local brain regions and produce relevant modulation on the behaviors (Ai et al., 2016, 2018). For example, TUS over frontal eye fields modulated the choice behavior in the macaque monkey (Kubanek et al., 2020), TUS over visual cortex induced phosphene phenomenon (Lee et al., 2016b), TUS over somatosensory cortex elicited tactile sensations in the hands and improved sensory discrimination capability (Lee et al., 2015; Legon et al., 2014; Liu et al., 2021), TUS over right anterior insula/frontal operculum and dorsal ACC reduced parasympathetic fear responses and emotional distraction interference on performance (Fini and Tyler, 2020a, 2020b), and TUS over primary motor cortex altered motor cortical excitability, which was reflected by motor evoked potential (MEP) measurement (Fomenko et al., 2020; Gibson et al., 2018; Legon et al., 2018b).

Previous studies have also tried to examine the repetitive TUS (rTUS) effect on brain regions (Blackmore et al., 2019; Pasquinelli et al., 2019). In animals, 40-s rTUS on the prefrontal cortex and supplementary motor area of primates altered functional connectivity (Verhagen et al., 2019). Besides, 20-s rTUS on the oculomotor area modulated the brain activity and relevant behaviors lasting for 20 min in non-human primates (Pouget et al., 2020). In deeper brain regions, such as the anterior cingulate cortex (ACC), 40s rTUS changed functional connectivity and impaired translation of counterfactual choice values into actual behavioral change (Fouragnan et al., 2019). Yang et al. (2021) observed bidirectional and state-dependent modulation effects when 30s rTUS was applied to Area 3a/3b, shown as different degrees of BOLD signal changes across off-target regions.

However, although the rTUS aftereffects are explored in non-human primates, the aftereffects of rTUS on the human cortex remain unelucidated. In human studies, 30s TUS on the right inferior frontal gyrus can improve mood and alter functional connectivity related to mood regulation (Sanguinetti et al., 2020); 15s rTUS on the frontal-temporal cortex induced mood improvement (Hameroff et al., 2013). Cain et al. investigated the effect of a 10-min session of rTUS on the left globus pallidus in humans and observed decreased blood oxygenation level-dependent (BOLD) signals in local and distal brain regions (Cain et al., 2021). However, previous studies mainly aim to investigate cognitive brain function change but not to explore the cortical plasticity change and the duration of aftereffects, which are essential for the clinical translation of rTUS intervention.

Motor cortex plays a vital role in the neural circuits for motor inhibitory control, involving both input and output of behavioral responses (Bari and Robbins, 2013; Chambers et al., 2009). The motor cortex drives the basal ganglia to prepare the specific motor response, including motor initiation and termination. Then, the basal ganglia region transmits the message back to the motor cortex through the thalamus (Nambu et al., 2002; Calabresi et al., 2014; Freeze et al., 2013). The impaired motor cortex plasticity can affect behavioral performance, such as motor learning (Huang et al., 2017). Sung et al. (2013) indicated that inhibitory repetitive TMS (rTMS) over the contralesional primary motor cortex (M1) and excitatory intermittent theta-burst stimulation over the ipsilesional M1 can facilitate motor performance in patients with chronic hemiplegic stroke (Sung et al., 2013). However, a larger randomized sham-controlled trial of 1-Hz rTMS over the contralesional M1 in patients with stroke showed no statistically significant improvement in the motor function compared with the sham group (Harvey et al., 2018). This divergence could be due to the different rTMS protocols (inhibit contralesional M1 and excite ipsilesional M1 simultaneously in Sung et al., 2013, whereas only inhibit contralesional M1 in Harvey et al., 2018), suggesting that inhibiting the contralesional side alone may not be powerful enough to induce motor function improvement. Therefore, the facilitation of the M1 cortex may enhance motor function and improve inhibitory control. In this study, we examined whether rTUS intervention to M1 can enhance the inhibitory control function in the behavioral task.

In the present study, we applied a new rTUS protocol. The current parameters have a long total rTUS duration (15 min) but a small total on-state duration (2.647 s). To ensure the safety of the current rTUS parameters, the ultrasound intensity is relatively low, the duty cycle is smaller (DC = 5%), and the inter-stimulus interval is larger (ISI = 8s) compared with previous studies (Table S1). With the rTUS protocol, we aim to produce clear aftereffects of rTUS on human motor cortex physiology and behavior on healthy participants and understand the time course of the evoked cortical plasticity. The motor inhibitory control task (Stop-signal task, SST) (Verbruggen et al., 2019) was used to reflect the effect of rTUS on the motor cortex at the behavioral level. We hypothesize that the rTUS stimulation to the left-M1 (l-M1) region could enhance cortex excitability and related inhibitory control function in healthy participants and produce a lasting neuromodulation effect. To test the hypothesis, we conducted an experiment to measure the single-pulse TMS-evoked MEPs and stop-signal reaction time pre- and post-rTUS. We expected to observe increased MEP amplitudes and reduced stop-signal reaction time in the SST task at post-rTUS compared with baseline at pre-rTUS, which will be the evidence to support our hypotheses. The results of this experimental study would provide the rationale for the application of rTUS as an intervention for brain disorders.

## Results

### Clinical information

**Figure 1:**
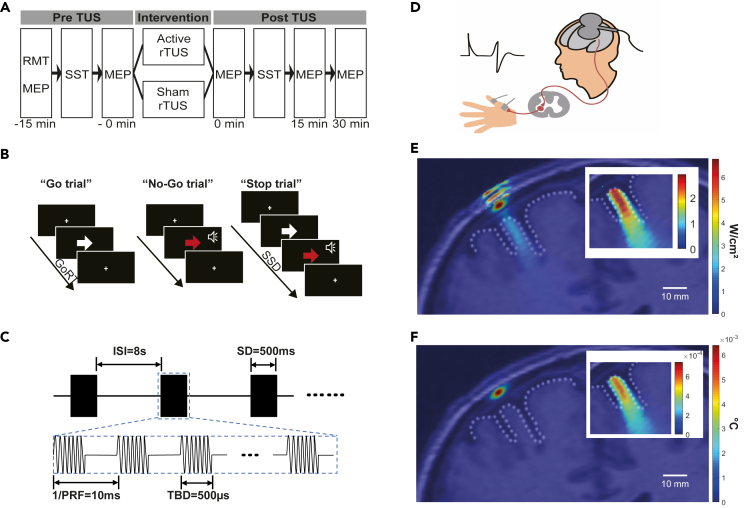
Study design and experiment setup (A) rTUS stimulation procedure. The RMT, two baseline motor-evoked potential (MEP), and SST were measured before active- and sham-rTUS intervention. After the intervention, MEPs at three time points with an interval of 15 min and SST were obtained. (B) Illustration of the Stop-signal task. (C) Schematic diagram of pulsed ultrasound waves and associated rTUS parameters used. (D) The illustration of MEP invoked by single-pulse TMS stimulation to left M1. RMT at the left M1 region was measured, and the MEP was recorded from the abductor pollicis brevis (APB) muscle on the right hand. (E) The simulated intracranial I_SPPA_ with a linear scale. The parameters of the simulation were as follows: fundamental frequency: 500 kHz; Grid points: 368 × 649; points per wavelength: 5; I_SPPA_ at the target position: 2.846 W/cm^2^. The inset figure shows the simulated I_SPPA_ at the brain cortex. (F) Map of the maximum simulated temperature after intermittent rTUS stimulation. The maximum simulated temperature increased 6.4 × 10^−3^°C in the skull and 6.1 × 10^−4^°C in the brain. The initial temperature is 37°C. The inset figure shows the simulated temperature at the brain cortex. The primary motor cortex cortical surface is shown with a dotted line. See also Tables S1, S2, and S3 and Figure S2.

In a crossover study design, 15 min of active- and sham-rTUS was applied over the l-M1 of the same group of subjects (Figures 1A–1D). No significant differences were observed in resting motor threshold (RMT), sleep quality, alcohol, cigarette use, anxiety, depression, impulsivity scores between the active-rTUS visit, and sham-rTUS visit (Table 1).

**Table 1:** Paired Student's t test in MEP amplitude and Wilcoxon-signed rank test in clinical information between sham-rTUS intervention and active-rTUS intervention

	Active (n = 24)	Sham (n = 24)	Statistics	
	Mean (SD)	Mean (SD)	T(23)	*p*
120% RMT	46.58 (7.07)	44.92 (7.22)	1.820	0.064
Baseline MEP1	0.96 (0.66)	0.93 (0.70)	0.189	0.852
Baseline MEP2	0.96 (0.67)	0.93 (0.67)	0.283	0.780
			**Z**	***p***
AUDIT	2.67 (6.35)	5.25 (7.92)	1.892	0.058
FTND	2.75 (1.73)	2.83 (1.97)	1.000	0.317
PSQI	4.96 (1.43)	4.88 (1.54)	−0.338	0.735
BAI	2.63 (5.08)	1.75 (2.66)	1.343	0.179
BDI	24.67 (4.03)	25.79 (4.52)	−0.598	0.550
BIS_noplan	25.42 (6.29)	24.08 (7.82)	−1.870	0.061
BIS_motor	21.17 (5.62)	22.42 (6.70)	0.522	0.602
BIS_attention	25.58 (6.39)	25.50 (6.22)	−0.880	0.379
BIS	24.06 (3.54)	24.00 (5.56)	−1.431	0.152

MEP1, MEP amplitude at 15 min before rTUS; MEP2, MEP amplitude at 0 min before rTUS; AUDIT, Alcohol Use Disorders Identification Test; FTND, Fagerstrom Test of Nicotine Dependence; PSQI, The Pittsburgh Sleep Quality Index; BAI, Beck Anxiety Inventory; BDI, Beck Depression Inventory; BIS, Barrett Impulsiveness Scale-11.

### The safety of the current rTUS protocol

To assess the safety of the current rTUS protocol, we first conducted measurements and simulations. Under the set parameters, the spatial-peak temporal-average intensity measured in water by hydrophone (NH1000 Needele Hydrophone-1.0mm, Precision Acoustics, UK) was I_SPTA_ = 0.403 W/cm^2^, the spatial-peak pulse average intensity was I_SPPA_ = 8.053 W/cm^2^, and mechanical index (MI) was 0.696. The extracranial ultrasound in simulation was I_SPTA_ = 0.401 W/cm^2^, I_SPPA_ = 8.020 W/cm^2^, and MI = 0.694; the intracranial ultrasound in simulation was I_SPTA_ = 0.142 W/cm^2^, I_SPPA_ = 2.846 W/cm^2^, MI = 0.420 (Figures 1E and S2, Table S3). The simulated results were overlaid on anatomical images in Figures 1E and 1F. The ultrasound intensity and MI in the current study are lower than the US Food and Drug Administration (FDA) guidelines for diagnostic ultrasound, which are defined as derated I_SPTA_ ≤ 720 mW/cm^2^, and either MI ≤ 1.9 or derated I_SPPA_ ≤ 190 W/cm^2^ (FDA, 2017). The maximum temperature increase after intermittent stimulation was 6.4 × 10^−3^°C in the skull and 6.1 × 10^−4^°C in the brain with the initial temperature set as 37°C (Figure 1F). The maximum simulated temperature increase of 2.65-s continuous stimulation was 0.134°C in the skull and 9.1 × 10^−3^°C in the brain. These results suggest that the ultrasound dosage used for neuromodulation induced slight temperature changes in the skull and brain. Besides, no adverse reactions were reported in the experiment, which also confirmed the safety of the current pulsing schemes.

### The aftereffects of rTUS intervention

**Figure 2:**
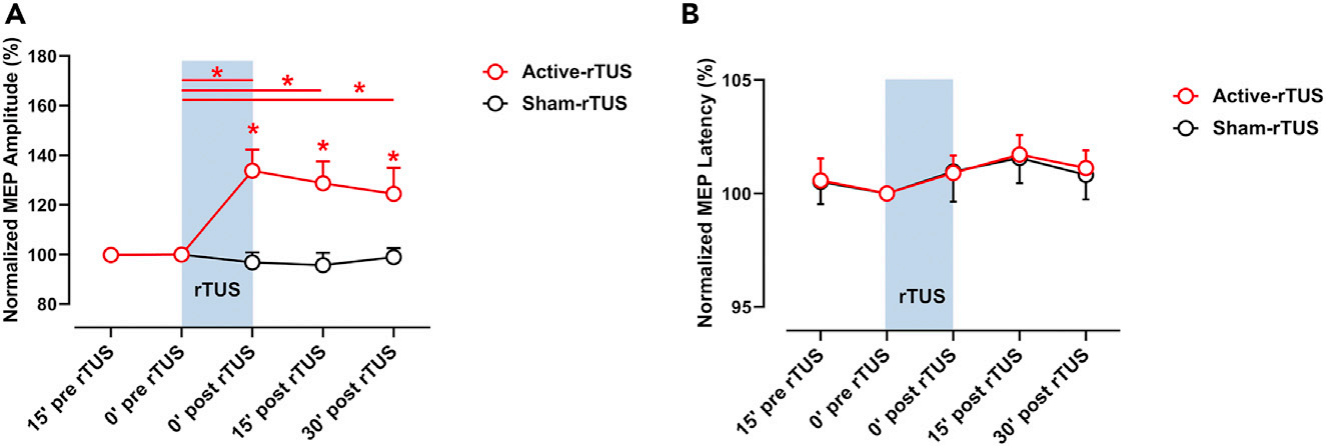
The normalized MEPs before and after active- and sham-rTUS intervention (A) The active-rTUS intervention but not sham-rTUS intervention results in potentiation of MEPs. (B) The latencies of MEPs in active- and sham-rTUS intervention, respectively. Red horizontal line: significant differences between the two time points in active-rTUS intervention. Blue rectangle: the period when rTUS intervention applied. Data are represented as mean ± SEM. Asterisk (∗) represents FDR corrected p value < 0.05 (Wilcoxon-signed rank test). See also Figure S1.

To investigate the aftereffects of rTUS intervention, we analyzed the MEP amplitudes at five time points. After 15 min active-rTUS intervention, MEPs were potentiated for more than 30 min (0 min post-rTUS: Z = −3.295, r = −0.476, FDR corrected p = 0.003, BF_10_ = 234.060; 15 min post-rTUS: Z = −2.857, r = −0.412, FDR corrected p = 0.009, BF_10_ = 17.712; 30 min post-rTUS: Z = −2.400, r = −0.346, FDR corrected p = 0.020, BF_10_ = 4.392; Figure 2A), when compared with baseline MEP of active-rTUS. Bayesian statistics showed substantial to strong evidence in favor of the research hypothesis of higher MEP amplitudes after active-rTUS intervention compared with baseline, whereas there were no significant changes in sham-rTUS intervention condition (0 min post-rTUS: Z = −0.913, r = −0.132, FDR corrected p = 0.542, BF_10_ = 0.282; 15 min post-rTUS: Z = −1.430, r = −0.206, FDR corrected p = 0.262, BF_10_ = 0.426; 30 min post-rTUS: Z = −0.395, r = −0.057, FDR corrected p = 0.923, BF_10_ = 0.231). Bayesian statistics support the null hypothesis that MEP amplitudes did not show differences after sham rTUS intervention. The MEPs of active-rTUS intervention showed significant higher amplitude when compared with sham-rTUS intervention condition at three time points (0 min post-rTUS: Z = −3.400, r = −0.491, FDR corrected p = 0.004, BF_10_ = 64.169; 15 min post-rTUS: Z = −2.514, r = −0.363, FDR corrected p = 0.018, BF_10_ = 24.229; 30 min post-rTUS: Z = −2.000, r = −0.289, FDR corrected p = 0.046, BF_10_ = 1.677). Bayesian statistics suggested strong evidence for higher MEPs after active-rTUS intervention compared with MEPs after sham-rTUS intervention at 0 min post-rTUS and 15 min post-rTUS, and only ambiguous evidence at 30 min post-rTUS. In terms of the MEP latency, there were no significant differences following active-rTUS or sham-rTUS (Figure 2B).

### rTUS on motor inhibitory control

**Figure 3:**
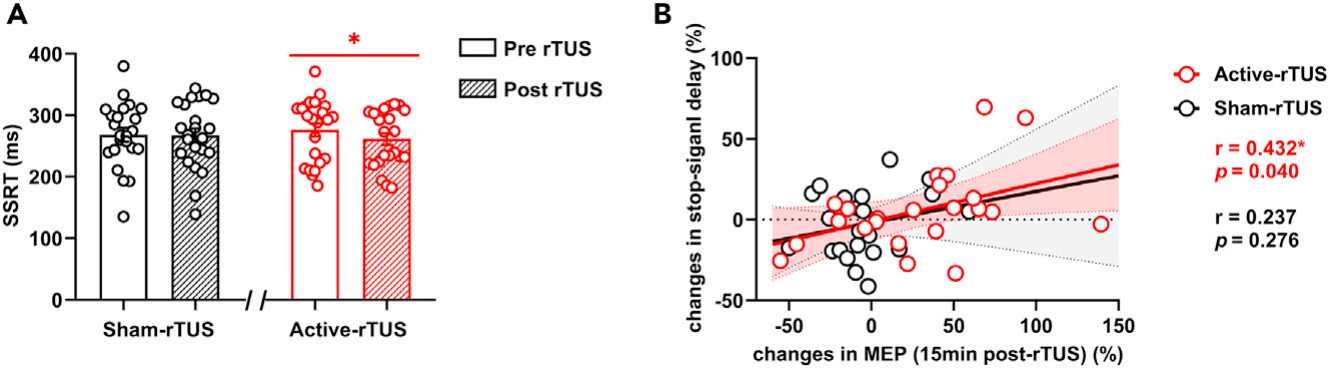
The results of the stop-signal task (A) Active-rTUS intervention but not a sham-rTUS intervention results in reduced stop-signal reaction time (SSRT). Data are represented as mean ± SEM. (B) The changes in stop-signal delay showed a positive correlation with changes in MEP (15 min post-rTUS) in active-rTUS but not sham-rTUS. Dashed curve and dash area: 95% confidence intervals. Asterisk (∗) represents FDR corrected p value < 0.05 (paired sample t test and Pearson's correlation analysis).

To demonstrate the effects of rTUS on motor inhibitory control, we analyzed the reaction time and reaction accuracy of the SST. The stop-signal reaction time (SSRT) did not show significant main effect of rTUS intervention (F (1,21) = 2.569, η_p_^2^ = 0.109, p = 0.124). No carryover effect was observed (F (1,21) = 0.065, η_p_^2^ = 0.003, p = 0.802), which indicates that the washout period of 7 days post the first rTUS visit is adequate. We then applied paired-sample t test to measure the SSRT before and after active- and sham-rTUS interventions and found that SSRT displayed significantly shorter SSRT (improved motor inhibitory control ability) after the active-rTUS intervention compared with baseline SSRT (t (22) = 2.355, FDR corrected p = 0.042) but showed no significant changes in the sham-rTUS intervention (t (22) = -0.747, FDR corrected p = 0.463) (Figure 3A). In terms of Go reaction time (GoRT) and stop-signal delay (SSD), no differences were observed. No significant differences were observed in Go trial accuracy, No-Go trial accuracy, and Stop trial accuracy between active-rTUS intervention and sham-rTUS intervention at baseline and post intervention.

### The carryover effect of rTUS

To explore the potential rTUS carryover effect, we analyzed the pre-rTUS values of the subjects that received active-rTUS intervention at visit 1. The MEP amplitudes, MEP ratio, MEP latency, RMT, SSRT, GoRT, and SSD as dependent variables were included in the analyses. There were no significant carryover effects in all variables (MEP amplitudes: F(1,24) = 0.330, p = 0.571, η_p_^2^ = 0.014; MEP ratio: F(1,24) = 0.117, p = 0.735, η_p_^2^ = 0.005; MEP latency: F(1,24) = 0.492, p = 0.490, η_p_^2^ = 0.020; RMT: F(1,24) = 0.995, p = 0.329, η_p_^2^ = 0.040; SSRT: F(1,24) = 0.154, p = 0.699, η_p_^2^ = 0.007; GoRT: F(1,24) = 0.024, p = 0.879, η_p_^2^ = 0.001; and SSD: F(1,24) = 0.000, p = 0.999, η_p_^2^ = 0.001), suggesting single-session rTUS did not cause long-term (more than 7 days) changes in the human brain.

### The correlation between behavioral and MEP alterations

Correlation analysis was conducted to explore the relationship between motor cortical potentiation and SST outcomes. The MEP changes at 15 min post-rTUS displayed a significant positive correlation with SSD changes (r = 0.432, p = 0.040) in the active-rTUS intervention but not in the sham-rTUS intervention (Figure 3B). No significant correlations between MEP changes and SST changes were observed at 0 min post-rTUS and 30 min post-rTUS.

### Individual differences in rTUS response

**Figure 4:**
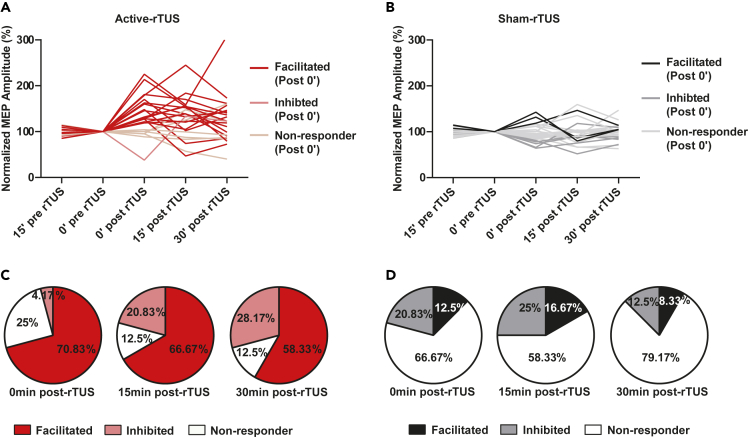
Individual response to rTUS in three time points (A) Individual MEPs of active-rTUS intervention. (B) Individual MEPs of a sham-rTUS intervention. (C) The percentage of participants who responded to active-rTUS intervention. (D) The percentage of participants who responded to sham-rTUS intervention. See also Figure S1.

To dissect the individual variation in response to rTUS intervention, the ranges of natural variability were defined as mean ± 2 SD of the normalized MEP at 15 min pre-rTUS (82.567–117.063), leading to the assignment of subjects into facilitation responder, no responder, and inhibitory responder subgroups (Figures 4A and 4B). Significant differences were observed between active-rTUS intervention and sham-rTUS intervention (0 min post-rTUS: χ^2^ = 17.012, p < 0.001; 15 min post-rTUS: χ^2^ = 14.409, p = 0.001; 30 min post-rTUS: χ^2^ = 22.236, p < 0.001). In the active-rTUS intervention, at the 0 min post-rTUS, around 70.83% (17 of 24) of the subjects were facilitation responders, 25% were no responders, and 4.17% were inhibitory responders. At 15 min post-rTUS, 66.67% of the subjects were facilitation responders, 12.5% were no responders, and 20.83% were inhibitory responders. At 30 min post-rTUS, 58.33% were facilitation responders, 12.5% were no responders, and 29.17% were inhibitory responders (Figure 4C). In sham-rTUS intervention, facilitation responder rates at three time points after rTUS were 12.5%, 16.67%, and 8.33%, respectively, whereas inhibitory responder rates at the three time points were 20.83%, 25%, and 12.50%, respectively (Figure 4D).

## Discussion

This study reported that rTUS can alter long-lasting cortex plasticity in human. These data confirm that one session of 15 min rTUS intervention over the left motor cortex can have considerable potentiation effects on both physiology and behavior in humans. The degree of motor cortical potentiation is comparable with other forms of TMS conditioning protocols, such as with 10 Hz repetitive stimulation or theta-burst stimulation. The findings therefore suggest the clinical potential of rTUS in the treatment for brain disorders.

This study observed at least 30 min cortex plasticity alteration and did not cause permanent change (no more than 7 days), suggesting an rTUS neural modulation effect and providing a clear effect duration for the current rTUS protocol. Several putative mechanisms may contribute to neuromodulation effects of rTUS: (1) membrane displacement associated with capacitance changes; (2) activation of mechanosensitive channels; (3) the sonoporation effect; (4) membrane waves coupling along the axon (Blackmore et al., 2019). Previous studies have shown that simultaneous TUS pulse reduced MEP amplitude, together with intracortical facilitation attenuation, increased or unchanged gamma-aminobutyric acid (GABA_A_)-mediated short-interval intracortical inhibition (SICI) (Fomenko et al., 2020; Legon et al., 2018b), suggesting for modulation on both excitatory and inhibitory components with TUS. These two studies focused on the immediate effect of 500-ms single-pulse TUS stimulation, whereas the present study investigated the lasting effect. It has been reported that the different states (during the task or resting state) are accompanied by different brain activations, leading to various TUS intervention effects (Yang et al., 2021). Nevertheless, the mechanism of excitatory effect and inhibitory effect of TUS is still not clear, and more studies are needed. Regarding the excitatory and inhibitory effects of TUS reported in different studies, Michael et al. proposed the neuronal bilayer sonophore model (Plaksin et al., 2014) and the neuronal intramembrane cavitation excitation model (Plaksin et al., 2016) to explain underlying mechanisms: ultrasound could selectively activate different cortical neuron subtypes (e.g., excitatory regular spiking [RS] pyramidal neuron, inhibitory fast-spiking [FS] cortical neurons, and inhibitory low-threshold spiking cortical neurons), and the network level neuromodulation outcomes (excitation-suppression) are determined by the interaction of the excitatory neurons and inhibitory neurons that are selectively activated or not by ultrasound stimulation. Recently, Yu et al. provided evidence that the excitatory RS neurons and inhibitory FS neurons had different responses to ultrasound and the specific neuron types could be preferentially targeted by tuning the pulse repetition frequency (PRF) of ultrasound pulse in *in vivo* anesthetized rodent brains (Yu et al., 2021). However, the precise mechanism of the rTUS modulation on the M1 region and its network effect requires further investigation.

The single session of active-rTUS on M1 improved behavioral inhibition in healthy subjects, demonstrated by reduced SSRT. Dysfunction in inhibitory control contributes to the behavioral abnormality in different psychiatric diseases, such as obsessive-compulsive disorder (Gilbert et al., 2004; Suppa et al., 2014), Tourette syndrome (Suppa et al., 2014), major depressive disorder (Cantone et al., 2017), and substance-use disorder (Shen et al., 2017). The present results therefore implied the clinical utility of rTUS in recovery from both neurological disorders (e.g., stroke) and impulsivity-related disorders. In this study, no subject reported any side effects and we did not observe a carryover effect after the active-rTUS intervention, which suggests that the current rTUS parameters could be safe for healthy human subjects.

No neuronavigation system was used to guide the positioning of the TMS coil and ultrasound transducer in the experiment, which may affect current MEP results due to the slight spatial shift of the TMS coil in multiple MEP measurements. To reduce the potential spatial shift, we measured the MEPs twice with 15-min intervals at baseline before rTUS application, which helps to evaluate the reliability of MEP measurement. MEP amplitudes at the first and second measurements were relatively stable in both groups. Besides, MEP amplitudes at baseline varied much less than at the post active-rTUS intervention and MEP amplitudes at baseline and the three measurements after sham-rTUS were relatively stable. Therefore, despite the lack of the neuronavigation, the current results suggest that the influence of potential spatial shift in multiple MEP measurements is in an acceptable range.

Auditory confound is one of the major concerns for TUS studies. Previous animal studies have shown that auditory confound can cause brain activation. Guo et al. (2018) observed that excitability changes induced by TUS in the brain can be partially due to an indirect effect of auditory stimulation. Besides, Sato et al. (2018) indicated that deafness could reduce the effects of TUS on the brain. However, some other studies have shown that TUS can directly affect brain activity without the auditory sense. Kubanek et al. (2016) showed that TUS affects neural activity in organisms that lack the auditory system. Morteza et al. showed that elimination of peripheral auditory pathway activation did not affect motor responses from ultrasound stimulation (Mohammadjavadi et al., 2019). In human studies, some studies have reported that subjects could perceive auditory sound when TUS was applied to human brain (Braun et al., 2020). TUS can lead to altered BOLD signal only in the stimulation region and concomitant behavioral changes, such as tactile sensations and visual phosphenes (Ai et al., 2018; Lee et al., 2016a, 2016b), which is hard to be explained by auditory activation. More importantly, pure tonal sounds or noise did not alter MEP amplitudes (Flöel et al., 2003). In the current study, we used a custom-made TUS system with a custom-made coupler filled with water to ensure proper coupling between the ultrasound transducer and scalp and thus avoid the air-caused ultrasonic energy attenuation. From reports of subjects after the TUS experiment, our TUS system does not produce sensible vibration or auditory sound during the intervention. Before choosing the transducer used in the current study, we have tested five different transducers and found that four of five transducers did not generate perceivable vibration and sound at the chosen TUS parameter. Besides, we observed rTUS induced significant effects on motor cortex physiology and behavior. Therefore, we concluded that the auditory confounds would not affect the neural and behavioral changes observed in the present study.

Safety is one major concern in applying TUS to the human brain. In this study, we proposed an rTUS protocol of low total dosage to ensure its safety. The rTUS protocol is with relatively low ultrasound intensity and a small total on-state duration compared with FDA guidelines and previous human brain studies. In this study, the spatial-peak pulse-average intensity of extracranial ultrasound measured in water by hydrophone was I_SPPA_ = 8.053 W/cm^2^, the corresponding spatial-peak temporal-average intensity was I_SPTA_ = 0.403 W/cm^2^, and the corresponding mechanical index was MI = 0.696. Our simulation results show that the extracranial ultrasound was I_SPPA_ = 8.020 W/cm^2^, I_SPTA_ = 0.401 W/cm^2^, and MI = 0.694; the intracranial ultrasound in simulation was I_SPPA_ = 2.846 W/cm^2^, I_SPTA_ = 0.142 W/cm^2^, MI = 0.420 (Table S3). The ultrasound intensity and MI used in this study are lower than most previous human studies (Table S1) and are below the FDA guidelines for diagnostic ultrasound (derated I_SPTA_ ≤ 720 mW/cm^2^, MI ≤ 1.9, I_SPPA_ ≤ 190 W/cm^2^) as well. To mimic the therapeutic intervention of TMS for patients with depression, we used an rTUS of 15 min total duration. To assure safety, we used a rather small duty cycle (DC = 5%) and large inter-stimulus interval (ISI = 8 s). We calculated the total on-state duration of TUS and found that the total on-state duration of TUS in the present study is 2.657 s, which is smaller than that of most previous studies (Table S1).

We also performed simulation on the temperature changes induced by rTUS with k-Wave (an acoustic simulation toolbox) based on the procedure in reference (Mueller et al., 2016; Rosnitskiy et al., 2019; Treeby and Cox, 2010). Results show that the maximum temperature increase was 6.4 × 10^−3^°C in the skull and 6.1 × 10^−4^°C in the brain with the initial temperature set as 37°C (Figure S2). These results suggest that temperature changes induced by rTUS are slight and thus the thermal effect of the rTUS would not induce brain injury. Note that the simulation results in previous human studies and experiment results in previous animal studies also indicated that the ultrasound dosage used for neuromodulation only induced slight temperature changes in skull and brain tissues (Lee et al., 2015, 2016a; Mueller et al., 2016).

The rTUS protocol was determined with the reference of safety data accumulated in previous animal studies. In our previous animal study, we observed that 2 weeks of TUS stimulation (15 min each day, 0.5 MHz, I_SPPA_ = 7.59 W/cm^2^, I_SPTA_ = 4.55 W/cm^2^, DC = 60%, TBD = 0.4 ms, ISI = 3 s, PRF = 1.5 kHz) had an antidepressant-like effect on depression model rats, and the histologic results showed no observable TUS-induced brain tissue injury (Zhang et al., 2019). In another study, Chu et al. reported that 120-s ultrasound stimulation (0.4 MHz, MI = 0.8, without microbubbles injected) to the forelimb region in the left primary somatosensory cortex of Sprague-Dawley rats did not induce blood-brain barrier (BBB) opening, brain tissue injury, and abnormal responses (in terms of both somatosensory evoked potentials and functional MRI-BOLD series) to the right forepaw electrical stimulation, whereas ultrasound stimulation of MI = 0.55 with microbubbles injected would yield BBB opening, brain tissue injury, and abnormal responses to the right forepaw electrical stimulation (Chu et al., 2015). The ultrasound dosage (I_SPTA_ and total on-state duration of TUS) in the present human study is smaller than that in our previous animal study. The MI = 0.696 (MI = 0.420 after attenuation by the skull in simulation, Table S3) in the present human study is smaller than the MI = 0.8 in the animal study (Chu et al., 2015). Furthermore, the human skull is much thicker than that of a rat and thus would have much more attenuation while ultrasound passes through it. Previous studies have reported that the brain lacks gas bodies (Dalecki, 2004) and thus the low-intensity ultrasound used in TUS would not induce inertial cavitation in brain tissues if no microbubbles are injected externally (Chu et al., 2015). So, with the reference of the animal studies mentioned above, we would argue that the rTUS protocol of this study would not induce inertial cavitation and tissue injury in the brain.

We further conducted carryover effect analysis after the active-rTUS for only subjects who received active-rTUS intervention at visit 1 and did not observe carryover effects in MEP amplitude, MEP ratio, MEP latency, RMT, SSRT, SSD, and GoRT, suggesting that the lasting effect (indicated by MEP amplitude) induced by single-session 15-min rTUS is less than 7 days. We measured the adverse reactions after each rTUS intervention (sham- and active-rTUS). No participants reported any kinds of side effects, including headache, neck pain, scalp pain, itch, feeling of burning, and mood changes. The carryover effect analysis and side effects reports both support that the 15-min rTUS protocol is safe for the human brain.

### Limitations of the study

There are several limitations for the present study. First, it is unknown if rTUS can cause long-term depression (LTD) of the motor cortex as well, as demonstrated by different rTMS protocols. Bidirectional modulation of synaptic plasticity with rTUS would produce controllable benefits in patients with brain disorders. Second, we did not combine rTUS with aftereffects measurement using neuroimaging methods or other synaptic measures (e.g., SICI). These measurements may provide mechanistic insights in neurochemical component changes following rTUS. Third, the finding at the motor cortex may not be generalized to other cortical regions. More investigations are required to prove the neuromodulation effects of rTUS on live human cortex. Last, MEP amplitudes could be affected by the inaccurate positioning of the TMS coil during MEP measurement as the navigator was not applied, although we applied twice baseline MEP measurements to ensure the reliability. To make the results more accurate, the navigation-guided TMS and rTUS are recommended in the further study.

In conclusion, the present study developed a method of delivering rTUS to the human cortex, which is safe and capable of producing a large effect on motor cortex plasticity and behavior. These findings suggest a considerable potential of rTUS in cortical plasticity modulation and clinical intervention for impulsivity-related disorders.

## STAR★Methods

### Key resources table

**Table:** 

REAGENT or RESOURCE	SOURCE	IDENTIFIER
**Deposited data**		

Motor-evoked potential data	This manuscript	https://osf.io/5qv4a/
Subjects’ characteristics	This manuscript	https://osf.io/n7934/
Stop-signal task data	This manuscript	https://osf.io/5q3cz/

**Software and algorithms**		

SPSS	IBM SPSS Statistics	https://www.ibm.com/products/spss-statistics
JASP	JASP	https://jasp-stats.org/
Matlab	Mathworks	www.mathworks.com/products/matlab.html
E-prime	Psychology Software Tools	https://pstnet.com/products/e-prime/

**Other**		

Clinical trial register number	ChiCTR2000039290	http://www.chictr.org.cn/showproj.aspx?proj=62963

### Resource availability

#### Lead contact

Further information and requests should be directed to the lead contact, Dr. Junfeng Sun (jfsun@sjtu.edu.cn).

#### Materials availability

This study did not generate new unique reagents.

### Experimental model and subject details

A total of 24 male healthy subjects (age 31.88±9.63 years) were recruited and randomly assigned (with computer-generated number sequence) into sham- or active-rTUS in their two visits. Subjects with sleep deprivation, neurological disorder, major medical illness, history of mental disorders, epilepsy, cardiovascular complications, implemented artificial cochlea, and other TMS contraindications were excluded. All participants were right-handed. As the mood states, sleep quality and the use of alcohol and cigarette could affect cortical plasticity (Conte et al., 2008; Khedr et al., 2021; Oathes et al., 2008; Player et al., 2013; van der Werf et al., 2010), the Pittsburgh Sleep Quality Index (PSQI), Beck Anxiety Inventory (BAI), Beck Depression Inventory (BDI), Barrett Impulsiveness Scale-11 (BIS), alcohol use condition (AUDIT), smoking use condition (FTND) were collected at each visit before the rTUS intervention. Ethics approval (2020-60) was obtained from the research ethics boards of Shanghai Mental Health Center and local safety monitoring board. All participants provided written informed consent.

### Method details

#### General aspects

This study was a single-blind cross-over design (Register No.: ChiCTR2000039290; Register date: October 22, 2020). The enrolment date of the first subject is October 25, 2020. The two groups (24 subjects in total) received either active-rTUS intervention or sham-rTUS intervention and then the alternative. The washout period was more than seven days to ensure that all rTUS effects vanished (Hameroff et al., 2013). The RMT was measured to determine the TMS intensity for MEP. Two MEP measurements were conducted before the rTUS stimulation with a time interval of 15 minutes. Then 15 minutes rTUS intervention (active or sham) was applied. After the intervention, MEPs were measured at 0 min post-rTUS, 15 min post-rTUS, and 30 min post-rTUS, respectively (Figure 1A). To test the behavior change, the SST was conducted before and after rTUS intervention (Figures 1A and 1B).

#### Stop-signal task

The stop-signal task (SST) is a widely used paradigm in studying response inhibition (Verbruggen et al., 2019; Verbruggen and Logan, 2008). The task includes three types of trials: 360 Go trials (75.0%), 40 No-Go trials (8.3%), and 80 Stop-signal trials (16.7%) (Figure 1B). In the Go trials, participants were required to press corresponding buttons in response to left/right black-colored arrows present on the screen for 1000 ms with their right hand as quickly as possible. Responses were made with either the index finger (for the left arrow) or the middle finger (for the right arrow). In the No-Go trials, a red-colored arrow was displayed on the screen for 1000ms with an audio tone, and participants were asked not to respond to the red arrow and tone. In the Stop-Signal trials, the left/right black-colored arrow first appeared and soon changed to the red-colored arrow with an audio tone after a Stop-Signal delay (SSD). Subjects were required to withhold their response action (i.e., no pressing buttons) in Stop-Signal trials. The SSD was continuously adjusted via a standard adaptive tracking procedure: SSD increases after each successful stop and decreases after each stop failure in a step of 50 ms. The initial estimate was 250 ms and the successful probability set around 50%. The outcome measure is stop-signal reaction time (SSRT), calculated by Go reaction time (GoRT) minus SSD. The SST was conducted before the rTUS and immediately after the rTUS. In this study, one participant did not correctly understand the behavioral task and the data was removed, therefore, 23 participants were included for SST analysis.

#### rTUS parameters

In the experiment, we used a novel rTUS protocol (duty cycle: DC=5%; tone-burst-duration: TBD=500 μs; sonication duration: SD=500 ms; the number of tone-bursts: NTB=50; the pulse repetition frequency: PRF=100 Hz; inter-stimulus interval: ISI=8 s; I_SPPA_=8.053 W/cm^2^; the stimulate duration was 15 min in total) (Figure 1C). For the current rTUS parameters, firstly, we refer to our previous TUS study on the depression rat model (Zhang et al., 2019). We observed that two weeks rTUS stimulation (15 min each day, 0.5MHz, I_SPPA_ = 7.59 W/cm^2^, I_SPTA_ = 4.55 W/cm^2^, DC=60%, TBD=0.4ms, ISI=3s, PRF=1.5kHz) to depression model rats had the antidepressant-like effect, and the histologic results of hematoxylin and eosin staining showed no observable TUS-induced brain tissue injury. The ultrasound dosage of the current study is smaller than this animal study. Besides, Chu et al. reported that 20 s ultrasound stimulation (0.4MHz, MI=0.8, without microbubbles injected) to the forelimb region in the left primary somatosensory cortex of SD rats did not induce BBB opening, brain tissue injury, and abnormal responses (in terms of both somatosensory evoked potentials and fMRI-BOLD responses) to the right forepaw electrical stimulation (Chu et al., 2015). The MI in our current human study (MI=0.696) is smaller than the MI=0.8 in this animal study. Then, to ensure the safety of the current rTUS parameters, we applied lower ultrasound intensity and a smaller duty cycle in the current study compared with previous human studies (Table S1). Moreover, the TUS parameters of previous studies are different from each other (Table S1). To compare the TUS duration in different studies, we defined a measure called on-state duration of TUS, which is the total time when ultrasound is delivered in all trails or stimulation duration, that is, the off-state in pulse sequence and the period of ISI was not taken into consideration. Resuls showed that in our 15 min rTUS, the on-state duration of TUS is only 2.647 s, which is smaller than that in most previous human studies (Table S1). So in terms of ultrasound intensity, MI, and on-state duration of TUS, the total TUS dosage in the current is smaller than in previous human studies, which ensures the safety of the rTUS protocol in the current study.

#### rTUS procedures

In the experiment, we used a custom-made TUS system with an immersion-type focused ultrasound transducer (V391-SU, Olympus NDT, Waltham, USA) operating at 0.5 MHz. The transducer was placed at the left M1 region about 1.3cm above the human brain, and the focal length is 3.8cm. A custom-made coupler filled with water was used to ensure proper coupling between the ultrasound transducer and scalp to avoid the air-caused ultrasonic energy attenuation. In the sham condition, the output of the TUS system was turned off. Therefore, no ultrasound was delivered to the head while the experimental procedure was maintained. Participants were provided earplugs and did not report any sensible differences between sham-rTUS condition and active-rTUS condition.

#### rTUS simulation

To simulate the acoustic propagation of TUS, the MRI slice of the M1 region from a typical male subject in another study was manually segmented into scalp, skull, brain tissue, and outer space filled with water (Rosnitskiy et al., 2019). The segmented tissue layers were regarded as a homogeneous tissue mask with material properties such as attenuation coefficient, sound velocity, and density shown in Table S2 (Mueller et al., 2016). At the TUS stimulation parameters used in the experiment, the estimated intracranial intensity and temperature of TUS were calculated by an acoustic simulation toolbox, k-Wave (Treeby and Cox, 2010). In the simulation, the acoustic source was defined to be a focused transducer driven by a sinusoid wave at 500 kHz with a target pressure of 419 kPa. The I_SPTA_ is related to the risk of thermal bio-effects, while I_SPPA_ and MI are related to the risk of cavitation (Pasquinelli et al., 2019).

Based on the maximum sound pressure amplitude, the volume rate of heating from focused ultrasound transducer was calculated by linear simulation and then used as the input of thermal simulation to calculate the maximum temperature value during ultrasonic stimulation (Treeby and Cox, 2010). Because ultrasound stimuli were divided into segments with longer intervals between each stimulus, the thermal effects of ultrasound decayed in the inter-stimulus interval, heat evoked by ultrasound stimulation is dissipated during intervals. The time interval between all ultrasonic stimuli within 15 min was removed, and the ultrasonic stimulus was changed to 2.65 s continuous release to calculate the maximum simulated temperature.

#### MEP procedures

MEP is a reliable and effective measurement index of cortical excitability, and enhanced MEP amplitude indicates increased cortical excitability and vice versa (Huang et al., 2017). Single-pulse TMS was delivered using a 75 mm outer diameter figure-of-8 coil and the MagPro stimulator (MCF-B65, MagVenture, Denmark). To examine the resting motor threshold (RMT) for each subject, electromyographic (EMG) signals were recorded from the right abductor pollicis brevis (APB) muscle with surface electrodes. EMG signals were amplified by Keypoint (Medtronic Co., Denmark) with a bandpass filter between 2 Hz and 10 kHz. The coil was held tangentially to the head, with the handle pointing backward and about 45° lateral to the midline. The area with the largest MEP was used as M1 for each subject. The resting motor threshold (RMT) was defined as the lowest intensity that produced an MEP of 50 μV (peak-to-peak) in at least five times out of ten trials in relaxed APB. Once the left M1 region was determined, we marked a redpoint there by marker pen to indicate the corresponding position, which was used as a landmark in the following positioning of the ultrasound transducer and TMS coil in rTUS and MEP measurements.

For each subject, RMT was first measured before sham and active-rTUS interventions. Twenty consecutive MEPs (5 s interval) were evoked by single-pulse TMS stimulation at five-time points (15 minutes before rTUS, 0 minute before rTUS, 0 minute after rTUS, 15 minutes after rTUS, 30 minutes after rTUS, Figure 1A) with 120% RMT intensity at the left M1 region, respectively. Values of MEP amplitude and latency were averaged for each time point. The rTUS procedure will be applied if there were no more than 15% differences between the two baseline values (15 minutes before rTUS, 0 minute before rTUS). If the fluctuation of MEP values at the two baselines is more than 15%, another 15 minutes will be waiting to obtain the third baseline value. If the last two baseline values are stable, then the MEP data will be collected. Otherwise, the participants will be excluded due to the failure to obtain a stable baseline. In this study, 24 participants were included for MEP measurements, and no participants were excluded. The MEP amplitude at 0 minute before rTUS was taken as 100% for each individual normalization.

### Quantification and statistical analysis

The Shapiro-Wilk test was used to test the normal distribution of clinical measurements and MEP data. The data of SSRT showed normal distribution, while clinical measurements and MEP amplitudes do not obey normal distribution. Therefore, the Wilcoxon Signed Rank Test was applied to test the differences between active-rTUS intervention and sham-rTUS intervention for clinical measurements and MEPs. The one-way ANOVA was applied to investigate the rTUS intervention effect and carry-over effect of SST, the sequences (sequence 1: active-rTUS, sham-rTUS; sequence 2: sham-rTUS, active-rTUS), intervention (active-rTUS, sham-rTUS), and subjects’ number were independent variables, values of post-rTUS intervention (SSRT, SSD, GoRT, Go-trial ACC, No-Go trial ACC, Stop-signal trial ACC) were dependent variables. Then, the paired-sample t-test was used to compare SST changes in different sessions (pre-rTUS, post-rTUS). To explore whether the rTUS intervention could cause the permanent change in the subjects’ motor cortex, the carry-over effect analysis was conducted only for the subjects that received active-rTUS intervention at visit 1. We applied One-way ANOVA to analyze the carry-over effect, the sequence that subjects received rTUS (visit 1: active-rTUS, visit 2: sham-rTUS) was as the independent variable and the pre-rTUS values (MEP amplitude, MEP ratio, MEP latency, RMT, SSRT, SSD, and GoRT) were as the dependent variables. Pearson's correlation analysis was conducted to explore the relationship between behavioural performance and MEP amplitude. All the statistical analyses were performed in SPSS (IBM SPSS Statistics version 21). The Benjamini–Hochberg FDR method was conducted to correct multiple comparisons.

To improve the inference about rTUS effects, we conducted the Bayesian Wilcoxon Signed-Rank test (zero-centered Cauchy prior width, r=0.707, 5 chains of 1000 iterations). Bayes factors (BFs) quantify the probability of the data under a research hypothesis (H1) relative to the probability of the data under the null hypothesis (H0). BF below 0.33 indicates moderate evidence for the H0 over H1, between 1 and 3 suggests ambiguous evidence for the H1, between 3 and 10 suggests substantial evidence, above 10 indicates strong evidence, and above 100 indicates decisive evidence. All Bayes factors reported here represent the evidence for H1 relative to H0. All the Bayesian analyses were conducted using JASP v0.14.1.0 (van Doorn et al., 2021).

### Additional resources

This study is part of a clinical trial (Clinical trial register number: ChiCTR2000039290): http://www.chictr.org.cn/showproj.aspx?proj=62963.

## Data Availability

MEP amplitudes data (‘MEP_dataset.xlsx’), subjects’ characteristic (‘subjects information.xlsx’), and Stop-signal task data (‘SST_dataset.xlsx’) are freely available via an open-access data-sharing repository and are publicly available as of the date of publication. Accession numbers are listed in the key resources table. The simulation code would be provided upon reasonable request. Information is listed in the key resources table. Any additional information required to reanalyze the data reported in this paper is available from the lead contact upon request.

## Supplemental information

Document S1. Figures S1 and S2 and Tables S2 and S3

Table S1. Summary of previous TUS studies’ parameters, related to STAR methods
