# Supplementary material for: Transcranial ultrasound stimulation of the human motor cortex
Source: iScience. 2021 Nov 13;24(12):103429. doi: 10.1016/j.isci.2021.103429 (PMC8637484; doi:10.1016/j.isci.2021.103429)
Supplement: Document S1. Figures S1 and S2 and Tables S2 and S3 [file mmc1.pdf]

iScience, Volume *24*

Supplemental information

Transcranial ultrasound stimulation of the human motor cortex

Yi Zhang, Liyuan Ren, Kai Liu, Shanbao Tong, Ti-Fei Yuan, and Junfeng Sun

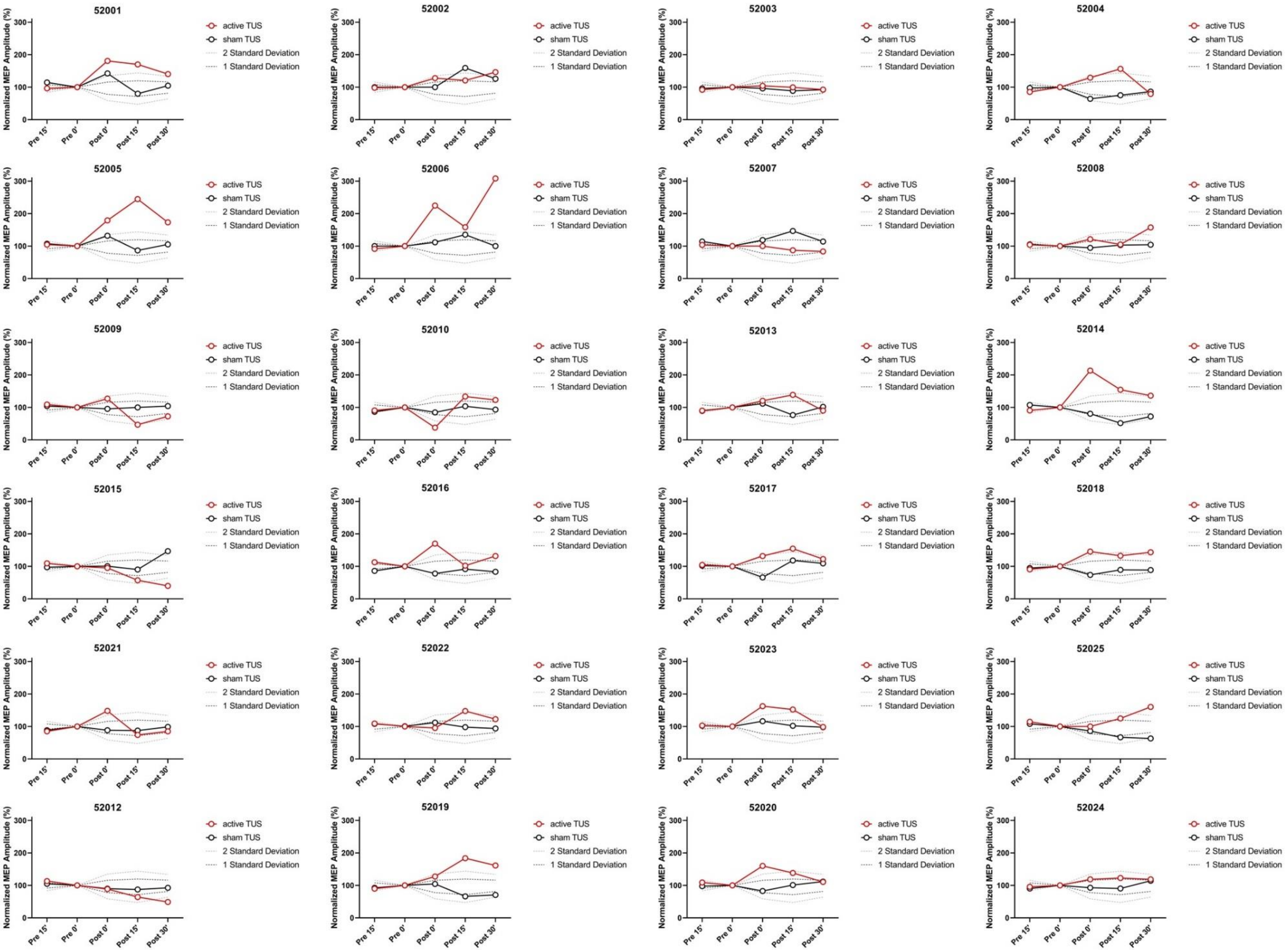

52001
52002
52003
52004
52005
52006
52007
52008
52009
52010
52013
52014
52015
52016
52017
52018
52021
52022
52023
52025
52012
52019
52020
52024
Normalized MEP Amplitude (%)
0
100
200
300
Pre 15'
Pre 0'
Post 0'
Post 15'
Post 30'
active TUS
sham TUS
2 Standard Deviation
1 Standard Deviation

Figure S1. Individual MEP amplitudes for rTUS in five time point, Related to Results Section. The black dash line: 1 standard deviation; the grey dash line: 2 standard deviations. Related to Figure 2 and 4.

Figure S2. The extracranial sound intensity in free water is measured by hydrophone (NH1000 Needle Hydrophone-1.0mm, Precision Acoustics, UK) **in water tank, and compared with the simulation results, Related to STAR Methods.** The extracranial (simulated) ultrasound was calculated by an acoustic simulation toolbox, k-Wave (Treeby and Cox, 2010). We used the MRI data of one typical subject in another MRI study to segment the scalp, skull, and brain tissues for numerical simulation. The segmented tissue layers were regarded as a homogeneous tissue mask with material properties such as attenuation coefficient, sound velocity and density shown in Table S2 (Mueller et al., 2016a). (A) The simulated I_{SPPA} in free water, I_{SPPA} at the focus is 8.020 W/cm^2. (B) The measured I_{SPPA} in free water, I_{SPPA} at the focus is 8.05W/cm^2, focal length: 39.7mm. Blue line: acoustic axial. Black line: the border of the full width at half maximum (FWHM) contour. White spot: markers at the centroid of the contours. Related to Figure 1.

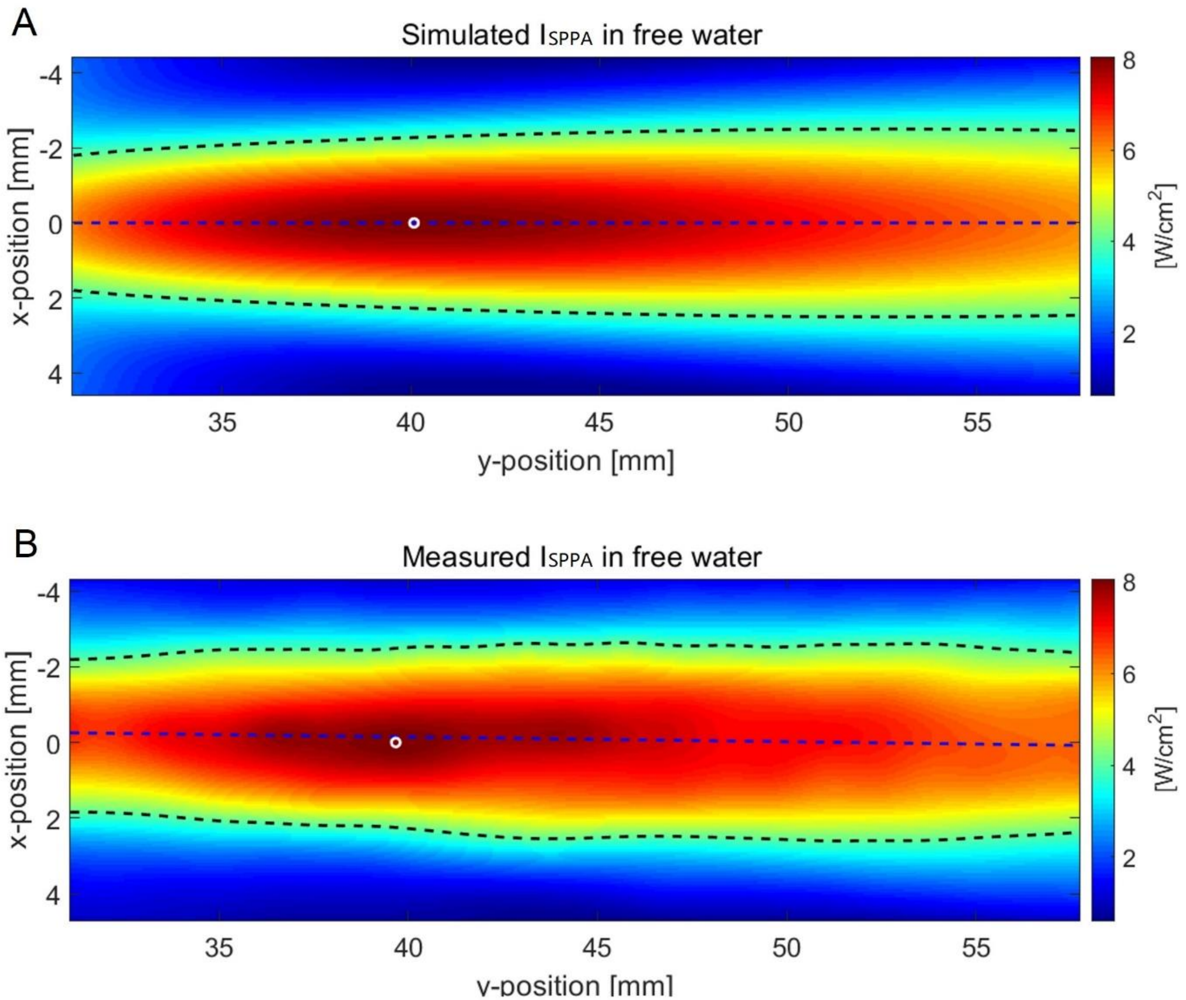

Table S2. Material properties for rTUS simulation, Related to STAR Methods.

Material	Speed of sound (m/s)	Density (kg/m^3)	Acoustic impedance (Pa·s/m)	Attenuation coefficient (Np/m)	Thermal conductivity (W·°C^{-1}·m^{-1})	Heat capacity (J·Kg^{-1}·°C^{-1})
Water	1503	999.5	1.48e6	0.02	0.595	4186
Scalp	1550	1030	1.60e6	0.92	0.528	3640
Skull	2300	1912	4.40e6	81	0.43	1440
Brain tissue	1550	1030	1.60e6	0.92	0.528	3640

CSF: cerebrospinal fluid. The extracranial (measured) ultrasound is measured in water by hydrophone (NH1000 Needle Hydrophone-1.0mm, Precision Acoustics, UK). The extracranial (simulated) ultrasound and the intracranial (simulated) ultrasound was calculated by an acoustic simulation toolbox, k-Wave (Treeby and Cox, 2010). We used the MRI data of one typical subject in another MRI study to segment the scalp, skull, and brain tissues for numerical simulation. The segmented tissue layers were regarded as a homogeneous tissue mask with material properties such as attenuation coefficient, sound velocity and density shown in Table S2 (Mueller et al., 2016b).

Table S3. Ultrasonic intensity parameters at the target position, Related to STAR Methods.

	5% DC, PRF = 100 Hz, SD = 500 ms			
	Pressure (KPa)	I_{SPTA} (W/cm^2)	I_{SPPA} (W/cm$^{2)}$	MI (unitless)
Extracranial (Measured)	492	0.403	8.053	0.696
Extracranial (Simulated)	491	0.401	8.020	0.694
Intracranial (Simulated)	297	0.142	2.846	0.420

DC: duty cycle; PRF: pulse repetition frequency; SD: sonication duration; I_{SPTA}: the spatial-peak temporal-average Intensity; I_{SPPA}: the spatial-peak pulse average intensity; MI: Mechanical Index.
